# Current cigarette smoking among in-school American youth: results from the 2004 National Youth Tobacco Survey

**DOI:** 10.1186/1475-9276-8-10

**Published:** 2009-04-03

**Authors:** Emmanuel Rudatsikira, Adamson S Muula, Seter Siziya

**Affiliations:** 1 Departments of Global Health, Epidemiology and Biostatistics, School of Public Health, Loma Linda University, Loma Linda, California, USA; 2 Division of Community Health, University of Malawi, College of Medicine, Blantyre, Malawi; 3 Department of Community Medicine, University of Zambia, School of Medicine, Lusaka, Zambia

## Abstract

**Background:**

Tobacco use is a leading cause of preventable morbidity and mortality. In the developed nations where the burden from infectious diseases is lower, the burden of disease from tobacco use is especially magnified. Understanding the factors that may be associated with adolescent cigarette smoking may aid in the design of prevention programs.

**Methods:**

A secondary analysis of the 2004 United States National Youth Tobacco Survey was carried out to estimate the association between current cigarette smoking and selected smoking-related variables. Study participants were recruited from middle and high schools in the United States. Logistic regression analysis using SUDAAN software was conducted to estimate the association between smoking and the following explanatory variables: age, sex, race-ethnicity, peer smoking, living in the same household as a smoker, amount of pocket money at the disposal of the adolescents, and perception that smoking is not harmful to health.

**Results:**

Of the 27727 respondents whose data were analysed, 15.9% males and 15.3% females reported being current cigarette smokers. In multivariate analysis, compared to Whites, respondents from almost all ethnic groups were less likely to report current cigarette smoking: Blacks (OR = 0.52; 95% CI [0.44, 0.60]), Asians (OR = 0.45; 95% CI [0.35, 0.58]), Hispanic (OR = 0.81; 95% CI [0.71, 0.92]), and Hawaii/Pacific Islanders (OR = 0.69; 95% CI [0.52, 0.93]). American Indians were equally likely to be current smokers as whites, OR = 0.98 [95% CI; 0.79, 1.22]. Participants who reported living with a smoker were more than twice as likely to smoke as those who did not live with a cigarette smoker (OR = 2.73; 95% CI [2.21, 3.04]). Having friends who smoked was positively associated with smoking (OR = 2.27; 95% CI [1.91, 2.71] for one friend who smoked, and OR = 2.71; 95% CI [2.21, 3.33] for two or more friends who smoked). Subjects who perceived that it was safe to smoke for one or two years were more likely to smoke than those who thought it was definitely not safe to do so. There was a dose-response relationship between age and the amount of money available to the respondents on one hand, and current smoking status on the other (p-value < 0.001).

**Conclusion:**

We found that White non-Hispanic adolescents were as likely to be current smokers as American Indians but more likely to be smokers than all other racial/ethnic groups. Older adolescents, increase amounts of pocket money, and perception that smoking was not harmful to health. The racial/ethnic differences in prevalence of smoking among America youth deserve particular exploration.

## Introduction

Tobacco use is a leading cause of preventable morbidity and mortality in the world [1,2]. Tobacco-related morbidity and mortality rank high especially in developed nations where the burden of disease from communicable or infectious diseases and maternal mortality is lower than in the developing nations of the world. Cigarette smoking causes serious illnesses among an estimated 8.6 million persons and approximately 440,000 deaths annually in the United States [3]. The prevalence of adult cigarette smoking in the United States has slightly decreased since the early 1990s from 24.7% in 1997 to 20.8% in 2006 [4,5]. This lack of a further decrease in cigarette use during this period might have been due to cuts in funding for comprehensive state programs for tobacco control and prevention by 20.3% from 2002 to 2006 while the tobacco-industry marketing expenditures nearly doubled from 1998 ($6.7 billion) to 2005 ($13.1 billion) [5,6].

Many of adult smokers start smoking as adolescents or young adults [7,8]. It is therefore imperative to prevent initiation of and maintenance of smoking among adolescents. Adolescent smoking is also associated with other unhealthy behaviors such as unsafe sexual intercourse [9,10], alcohol use [11,12] and truancy [13]. Cigarette smoking has also been described as a "gateway" substance towards illicit drug use among adolescents and young adults [14,15]. Prevention of adolescent smoking therefore potentially has short term as well as long-range benefits. The U.S. Department of Health and Human Services has used data on the prevalence of cigarette smoking among adolescents as an indicator of future burden of chronic disease in the country. Internationally, much of the data on global adolescent tobacco use come from the Global Youth Tobacco Survey [16-20].

The prevalence of tobacco use among in-school adolescents in the US using the 2004 National Youth Tobacco Survey (NYTS) have been reported before [21]. However this report did not assess factors that are associated with smoking in this group. We therefore used data from the United States to conduct further analyses to assess whether the following factors are associated with smoking among adolescents in the US: age; ethnicity; living with smoker in the same household; perception that it is alright to smoke for one to two years as long as one stops thereafter; smoking in best friends and the amount of pocket money at the disposal of the adolescent.

## Methods

This study was based on secondary analysis of the NYTS of 2004. A comprehensive description of the survey has been reported by the Centers for Disease Control and Prevention [21,22]. Of the 31,774 students who were eligible to participate in the study, 27,933 (88%) completed the survey (14,034 middle school students [grades 6–8], 13,738 high school students [grades 9–12], and 161 students unclassified with respect to grade).

Data were weighted with the intention to adjust for design effects (non-independence) resulting from sampling within clusters in order to produce estimates that should be nationally representative. SUDAAN version 9.0 (Research Triangle Institute, Research Triangle Park, North Carolina, USA) was used for data analysis. Prevalence of current smoking and selected relevant socio-demographics were obtained. Current smoking was defined as having smoked a cigarette at least once in the last 30 days. Bivariate and multivariable logistic regression analyses were conducted to assess factors that are associated with current cigarette smoking. The explanatory variables that were assessed are: age; ethnicity; living with smoker; perception that it is alright to smoke for one to two years as long as one stops thereafter; smoking in best friends and the amount of pocket money at the disposal of the adolescent. For the purpose of these analyses, complete data were available for 27,727 study participants.

## Results

### Characteristics of the study participants

Data for 27,727 students who participated in the study were analyzed of whom 13,958 (50.4%) were males and 13,769 (49.6%) were females. The median age was 14 years (Q_1 _= < 16 years, Q_3 _= 13 years).

### Prevalence of cigarettes smoking

Table 1 indicates that of the 27,727 participants, 15.9 males and 15.3% females reported being current cigarette smokers. Whites had the highest rate of smoking (17.6%) while Asians had the lowest (9.3%).

**Table 1:** Prevalence of current smoking among in-school American youth

Characteristic	Total	Males	Female
Age (years)			

Total	15.7	15.9	15.3

≤ 12	4.0	4.3	3.7

13	9.3	10.0	8.6

14	13.3	14.6	12.0

15	16.7	17.3	16.0

16+	25.7	25.4	25.8

Ethnicity			

White	17.6	18.7	16.5

Black or African American	9.3	8.8	9.8

Asian	9.0	7.7	9.7

Hispanic	15.0	13.8	16.4

American Indian/Alaska Natives	17.0	15.7	18.3

Native Hawaiian/Other Pacific Islander	16.3	13.0	18.2

Lived with a cigarette smoker			

No	10.0	9.8	10.

Yes	24.0	24.9	23.1

Closest friends smoked cigarettes			

None	12.1	13.5	10.6

One	33.4	32.9	34.0

Two or more	41.0	42.6	40.1

Perception that it is safe to smoke for only a year or two, if quit after that			

Definitely not	7.7	8.4	6.7

Probably not	27.9	31.3	24.5

Probably yes	46.2	49.0	44.1

Definitely yes	51.2	45.2	53.3

Pocket money ($/week)			

None	7.9	8.7	7.0

1–10	9.4	10.6	8.0

11–20	15.4	15.9	14.8

21–50	21.3	21.7	20.8

51–100	25.5	27.6	23.8

>100	33.3	31.8	32.7

Current smoking defined as having smoked cigarette at least one day in the last 30 days

### Factors associated with being a current cigarette smoker

Table 2 indicates that for both males and females, age and ethnicity, living with a cigarette smoker, having best friends cigarette smokers, perception that it is safe to smoke for only a year or two, and pocket money were associated with current cigarette smoking.

**Table 2:** Unadjusted Odds ratios (ORs) and 95% confidence intervals [CI) of factors associated with current smoking among in-school American youth

Characteristic	Total OR [95% CI]	Male	Female
Age (years)			

Total			

≤ 12	1.00	1.00	1.00

13	2.44 [2.03, 2.94]	2.49 [1.93, 3.21]	2.49 [1.90, 3.27]

14	3.67 [3.07, 4.39]	3.85 [3.01, 4.93]	3.59 [2.76, 4.67]

15	4.78 [4.01, 5.71]	4.71 [3.68, 6.01]	5.02 [3.88, 6.49]

16+	8.26 [7.06, 9.67]	7.65 [6.15, 9.51]	9.20 [7.30, 11.59]

Ethnicity			

White	1.00	1.00	1.00

Black or African American	0.48 [0.43, 0.54]	0.42 [0.35, 0.50]	0.55 [0.47, 0.65]

Asian	0.46 [0.37, 0.57]	0.36 [0.26, 0.50]	0.54 [0.41, 0.72]

Hispanic	0.83 [0.75, 0.92]	0.70 [0.61, 0.80]	0.99 [0.86, 1.14]

American Indian/Alaska Natives	0.97 [0.82, 1.15]	0.81 [0.63, 1.05]	1.14 [0.91, 1.43]

Native Hawaiian/Other Pacific Islander	0.91 [0.73, 1.15]	0.65 [0.45, 0.95]	1.13 [0.84, 1.52]

Lived with a cigarette smoker			

No	1.00	1.00	1.00

Yes	2.83 [2.60, 3.08]	3.06 [2.71, 3.46]	2.63 [2.33, 2.97]

Closest friends smoked cigarettes			

None	1.00	1.00	1.00

One	3.63 [3.15, 4.19]	3.15 [2.55, 3.88]	4.36 [3.58, 5.31]

Two or more	5.03 [4.27, 5.95]	4.76 [3.66, 6.19]	5.69 [4.60, 7.05]

Perception that it is safe to smoke for only a year or two, if quit after that			

Definitely not	1.00	1.00	1.00

Probably not	4.65 [4.21, 5.13]	4.97 [4.34, 5.68]	4.40 [3.80, 5.09]

Probably yes	10.32 [9.01, 11.82]	9.50 [6.42, 12.64]	10.71 [8.85, 12.96]

Definitely yes	12.63 [10.42, 15.30]	10.47 [8.59, 12.76]	15.49 [12.17, 19.71]

Pocket money ($/week)			

None	1.00	1.00	1.00

1–10	1.21 [1.04, 1.41]	1.24 [1.01, 1.53]	1.14 [0.91, 1.44]

11–20	2.12 [1.82, 2.46]	1.99 [1.62, 2.44]	2.29 [1.83, 2.85]

21–50	3.15 [2.71, 3.66]	2.92 [2.37, 3.59]	3.47 [2.78, 4.32]

51–100	3.99 [3.37, 4.71]	4.02 [3.18, 5.06]	4.12 [3.24, 5.25]

>100	5.56 [4.46, 6.50]	4.91 [3.90, 6.18]	6.40 [5.14, 7.96]

Current smoking defined as having smoked cigarette at least one day in the last 30 days All groups other than "Hispanic" are non Hispanic

Table 3 indicates that findings from multivariate analysis remained unchanged. Compared to Whites, respondents from almost all ethnic groups were less likely to report current cigarette smoking: Blacks (OR = 0.52; 95% CI [0.44, 0.60]), Asians (OR = 0.45; 95% CI [0.35, 0.58]), Hispanic (OR = 0.81; 95% CI [0.71, 0.92]), and Hawaii/Pacific Islanders (OR = 0.69; 95% CI [0.52, 0.93]). American Indians were equally likely to be current smokers as whites, OR = 0.98 [95% CI; 0.79, 1.22].

**Table 3:** Adjusted odds ratios (AORs) and 95% confidence intervals [CI) of factors associated with current smoking among in-school American youth

**Characteristic**	**Total** **AOR (95% CI)**	**Male**	**Female**
Age (years)			

Total			

≤ 12	1.00	1.00	1.00

13	1.97 [1.57, 2.46]	1.91 [1.42, 2.58]	2.05 [1.47, 2.86]

14	3.28 [2.64, 4.08]	3.24 [2.41, 4.35]	3.35 [2.42, 4.64]

15	3.63 [2.92, 4.50]	3.63 [2.71, 4.86]	3.63 [2.62, 4.99]

16+	5.59 [4.57, 6.83]	5.62 [4.28, 7.37]	5.56 [4.14, 7.55]

Ethnicity			

White	1.00	1.00	1.00

Black or African American	0.52 [0.44, 0.60]	0.41 [0.34, 0.51]	0.70 [0.56, 0.87]

Asian	0.45 [0.35, 0.58]	0.32 [0.22, 0.46]	0.65 [0.46, 0.92]

Hispanic	0.81 [0.71, 0.92]	0.62 [0.51, 0.75]	1.12 0.93, 1.35]

American Indian/Alaska Natives	0.98 [0.79, 1.22]	0.75 [0.55, 1.03]	1.31 [1.00, 1.77]

Native Hawaiian/ Other Pacific Islander	0.69 [0.52, 0.93]	0.53 [0.33, 0.87]	0.89 [0.60, 1.31]

Lived with a cigarette smoker			

No	1.00	1.00	1.00

Yes	2.73 [2.46, 3.04]	2.97 [2.57, 3.43]	2.48 [2.13, 2.89]

Closest friends smoked cigarettes			

None	1.00	1.00	1.00

One	2.27 [1.91, 2.71]	2.18 [1.70, 2.80]	2.42 [1.91, 3.08]

Two or more	2.71 [2.21, 3.33]	2.87 [2.05, 4.02]	2.64 [2.11, 3.55]

Perception that it is safe to smoke for only a year or two, if quit after that			

Definitely not	1.00	1.00	1.00

Probably not	4.12 [3.67, 4.63]	4.54 [3.89, 5.3]	3.62 [3.04, 4.31]

Probably yes	9.35 [7.92, 11.04]	9.78 [7.64, 12.53]	8.86 [7.06, 11.11]

Definitely yes	11.82 [8.99, 15.54]	10.35 [6.35, 16.84]	12.21 [8.79, 16.98]

Pocket money ($/week)			

None	1.00	1.00	1.00

1–10	1.28 [1.06, 1.54]	1.32 [1.03, 1.69]	1.19 [1.00, 1.59]

11–20	1.78 [1.48, 2.13]	1.70 [1.34, 2.17]	1.88 [1.43, 2.47]

21–50	2.21 [1.84, 2.67]	2.06 [1.60, 2.66]	2.41 [1.83, 3.18]

51–100	2.47 [2.00, 3.08]	2.48 [1.85, 3.33]	2.46 [1.79, 3.39]

>100	2.89 [2.34, 3.56]	2.66 [1.97, 3.59]	3.17 [2.36, 4.25]

Current smoking defined as having smoked cigarette at least one day in the last 30 days All groups other than "Hispanic" are non Hispanic

Participants who reported living with a smoker were more than twice as likely to smoke as those who did not live with a cigarette smoker (OR = 2.73; 95% CI [2.21, 3.04]). Having friends who smoked was positively associated with smoking (OR = 2.27; 95% CI [1.91, 2.71] for one friend who smoked, and OR = 2.71; 95% CI [2.21, 3.33] for two or more friends who smoked). Subjects who perceived that it was safe to smoke for one or two years were more likely to smoke than those who thought it was definitely not safe to do so. There was a close-response relationship between age, and the amount of money available to the respondents on one hand and smoking on the other (p-value < 0.001).

## Discussion

We report in the current study an overall prevalence of current cigarette smoking among the studied cohort in 2004 of 15.7%, with lower smoking rates at lower ages from 4.0% in those 12 years old or younger to 25.7% in those 16 years or older. Males predominated in being smokers as has been shown in other studies [23-25]. However, in absolute terms, the difference in prevalence was rather minimal, 15.9% and 15.3% in males and females respectively. Our study also found that having a close friend who was a smoker, or living in a household with a smoker were both independently associated with being a current smoker among the adolescents. Previous studies have reported similar correlation between these characteristics and smoking [26-28].

In a prospective study on adolescent alcohol drinking reported by Fisher et al [29], having adults who drink in the home, underage sibling who drinks, peer who drinks, possession of or willingness to use alcohol promotional items, and positive attitudes toward alcohol were associated with an increased likelihood of alcohol initiation. The mechanisms operating in the case of alcohol i.e. role modeling, acceptability of a behavior within the home and among siblings and peers, easy access to a substance and positive attitude towards a behavior, may be operational in the case of smoking.

Due to the cross sectional nature of the data collection in the NYTS, it is not possible to ascribe causation or determine the exact sequence of events in an adolescents' smoking trajectory. It is plausible to consider that adolescents who befriend smokers are more likely to be influenced into smoking. It is equally plausible that adolescents who smoke are more likely to choose other smokers as their friends [30,31]. Adult smokers within the home are also less likely to discourage adolescents from smoking. Furthermore the easy availability of cigarettes when other people are smokers in the home may facilitate initiation and maintenance of smoking among the adolescents. Conley Thomson et al [32] have reported that household smoking bans limits smoking among adolescents. Adults who do not smoke themselves are more likely to discourage smoking in the home and elsewhere than smokers. This study also found that as age increases, the likelihood of being a current smoker also increases.

We also found that white-non Hispanic adolescents were more likely to be smokers than African-American, Asian-Americans and Hispanic. American Indians however were as likely as Whites to be smokers. We did not determine why this may be the case from the available data. However, a literature review report by Tauras [33] found that Hispanics and African Americans were more responsive to changes in cigarette prices than whites. Furthermore, one study that was reviewed reported that adolescent white males who smoked were responsive to changes in smoke-free air laws, while adolescent blacks who smoked were responsive to changes in youth access laws. This may suggest that different racial/ethnic groups may respond to different public health interventions.

In agreement to previous studies [23,24], perception that smoking was not harmful to health and having pocket money were associated with being a current smoker among adolescents. In an environment where adolescents may be legally employed to earn income from employment, it is possible that much of an adolescent's income may not be coming from parents. As such expecting parental supervision on adolescent spending may be particularly difficult. Also, it is possible that the amount of pocket money available to the adolescent may have just been a surrogate variable for the unmeasured time that the adolescent spends at work. Ramchand et al [34] have reported that the longer the hours an adolescent worked, the more likely she or he was to be a smoker.

Overall, 1 in 10 adolescents in the current study thought smoking was "cool", 1 in 3 owned an item with a tobacco brand, and exposure to pro-tobacco advertisements at gas stations, on the internet and smoking by characters in movies/television exceeded 40%. This amount of exposure should be seen in the light of evidence that media exposure to tobacco advertisements is associated with adolescent smoking [35-37].

### Limitations of the study

This study had several limitations. First, the results reported in this study were based on self-reports. Study participants may have intentionally mis-reported history of smoking or done so inadvertently. In addition, the levels of reliability and validity in the specific settings where data collection occurred may result in differential bias. Furthermore, self-reported history of smoking was not validated with laboratory markers such as exhaled carbon monoxide, and blood or urine cotinine [38-40]. Brener et al [41] however have reported high reliability of this methodology to assess adolescent health risky behaviors. However, in the design and administration of the surveys, various steps were taken to mitigate such bias, for example students were participated anonymously. This was aimed to prevent intentional mis-reporting for fear of reprisals from school officials. However, it is not possible to gauge how far the study participants completed the questionnaires as accurately as possible.

Smoking rates among students who were habitually absent may have been different from the rates among their counterparts who attended school regularly. The external validity of the results from the current study may therefore be limited to students who were not absent on the day of administration. It is however likely that absent students are more likely to be smokers than those present in schools [42,43], so our findings are likely to be underestimates.

Among persons aged 16 or 17 years in the United States, about 5% were not enrolled in a high school education and had not completed high school in 2000 [44]. The questionnaire was offered only in English, and so with the increase in non-US born adolescents who may have difficulties in comprehension, there may have been misreporting. Finally, the factors assessed in this study were limited to socio-demographic variables. Key psychosocial variables known to affect substance use such as perceived norms, outcome expectancies, ability to resist peer appeals (self-efficacy), depression, truancy and stress were not included.

## Conclusion

We found that White non-Hispanic adolescents were as likely to be current smokers as American Indians but more likely to be smokers than all other racial/ethnic groups. Older adolescents, increase amounts of pocket money and perception that smoking was not harmful to health were associated with being a smoker. There is need to explore the reasons why the prevalence of smoking among difference racial groups differ in United States.

## Abbreviations

GYTS: Global Youth Tobacco Survey; YRBS: Youth Risk Behavior Survey; NYTS: National Youth Tobacco Survey; US: United States.

## Conflict of interests

The authors declare that they have no competing interests.

## Authors' contributions

ER: conceived data analysis plan, conducted data analysis, and participated in the drafting of the manuscript. AM: participated in the interpretation of the results and drafting of the manuscript. SS: participated in the interpretation of the results and drafting of the manuscript. All authors read and approved the manuscript.
